# Heterospecific pollination by an invasive congener threatens the native American bittersweet, *Celastrus scandens*

**DOI:** 10.1371/journal.pone.0248635

**Published:** 2021-03-23

**Authors:** David N. Zaya, Stacey A. Leicht-Young, Noel B. Pavlovic, Mary V. Ashley

**Affiliations:** 1 Department of Biological Sciences, University of Illinois at Chicago, Chicago, Illinois, United States of America; 2 U.S. Geological Survey, Great Lakes Science Center, Chesterton, Indiana, United States of America; Instituto Nacional de Pesquisas da Amazonia, BRAZIL

## Abstract

Invasive plants have the potential to interfere with native species’ reproductive success through a number of mechanisms, including heterospecific pollination and hybridization. This study investigated reproductive interactions between a native North American woody vine (American bittersweet, *Celastrus scandens*) and an introduced congener (oriental bittersweet, *C*. *orbiculatus*). The decline of *C*. *scandens* in the eastern portion of its range is coincident with the introduction and spread of *C*. *orbiculatus*, and the two species are known to hybridize. The relationship between proximity and floral production of conspecific and heterospecific males on fertilization and hybridization rates was measured at a field site in northwestern Indiana, USA where both species occur and reproduce. We found that the invasive vine had an extreme advantage in both male and female floral production, producing nearly 200 times more flowers per staminate plant and 65 times more flowers per pistillate plant than the native. Using nuclear microsatellite DNA markers we found that hybridization rates were asymmetric; 39% of the *C*. *scandens* seeds tested were hybrids, compared to only 1.6% of *C*. *orbiculatus* seeds. The asymmetric hybridization rates were likely not solely due to greater abundance of *C*. *orbiculatus* pollen because experimental hand crosses revealed that *C*. *scandens* had a higher rate (41%) of heterospecific fertilization than *C*. *orbiculatus* (2.4%). We previously reported that few hybrids were observed in the wild, and hybrids had greatly reduced fecundity. Thus, in our system, the threat posed by heterospecific pollen is not replacement by hybrids or introgression, but rather asymmetric reproductive interference. Reproductive interference extended to distances as great as 100 meters, thus, efforts to conserve the native species must reduce its exposure to *C*. *orbiculatus* over a relatively large spatial scale.

## Introduction

Threats posed by invasive plant species to native ecosystems are a growing concern, with mounting evidence of increasing rates of invasion [1–3], disruptions of ecosystem function [1, 4–6], and reductions in native community diversity [5, 7–9]. Recent work has highlighted the potential of invasive plants to disrupt pollination systems and negatively impact reproductive success of native plants [10–12]. While much attention has focused on pollinator networks and pre-pollination dynamics in invaded systems [10, 11, 13], invasive species can also alter post-pollination dynamics. Threats posed by increased heterospecific pollination (also referred to as ‘interspecific’ pollen by some authors) and reproductive interference during invasions has been documented in several systems [14–17]. Two recent meta-analyses demonstrate that the threat posed by invasive plants through reproductive interference is potentially widespread. Arceo-Gómez and Ashman [12] found that invasive plants are more detrimental as heterospecific pollen donors than are native plants, and that the negative effects are greater when the invasive pollen donor and recipient are closely related. Burns et al. [18] found that invasives produce more than 250 times more flowers per plant compared to their native relatives. While Burns et al. [18] emphasize that this allows invasives to compensate for or avoid pollen limitation, an additional consequence could be increased heterospecific pollination of co-occurring natives that share their pollinators.

Reproductive interference by invasives can occur even without disruption of pollinator networks and visitation rates through heterospecific pollination. Pollen from an invasive species may reduce successful fertilization of natives by conspecific pollen [16]. This could occur through chemical or physical interference in the stigma by invasive pollen [19] or because the invasive pollen is simply much more abundant than native pollen [20]. Rejection of invasive pollen is especially difficult when the native and invasive species are closely related, such as congeners [12]. If heterospecific fertilization occurs, female reproductive effort may be wasted on fertilized ovules that rarely develop into viable seeds [21]. If viable hybrids survive, this may lead to decline of the native due to seed discounting and wasted reproductive effort [22]. Additionally, hybrids may be especially numerous, fit, or fecund, leading to “hybrid swarms” that exclude the native species [6, 23, 24]. Finally, continued asymmetric introgression may lead to genetic dilution of the native species’ gene pool [20, 25, 26].

In this study we investigated reproductive interactions between an invasive vine, *Celastrus orbiculatus* Thunb. and a native congener, *C*. *scandens* L. *Celastrus orbiculatus* was introduced to North America from Asia [27] and has subsequently spread across much of the native range of *C*. *scandens*. The two species are known to hybridize in controlled settings [28] and in the wild [29]. Hybrid individuals are uncommon in the wild, but a previous study found that all genetically identified hybrids had a *C*. *scandens* maternal lineage [29]. It has not been demonstrated that this asymmetric hybridization is linked to the decline of *C*. *scandens*, but declines are especially severe in parts of the eastern United States where the invasion is the oldest [30, 31]. While large-scale plant surveys can provide some insight into the dynamics of invasion and hybridization, fine-scale studies of invasive heterospecific pollen transfer are needed to directly assess impacts on native plants [12, 19].

**Fig 1:**
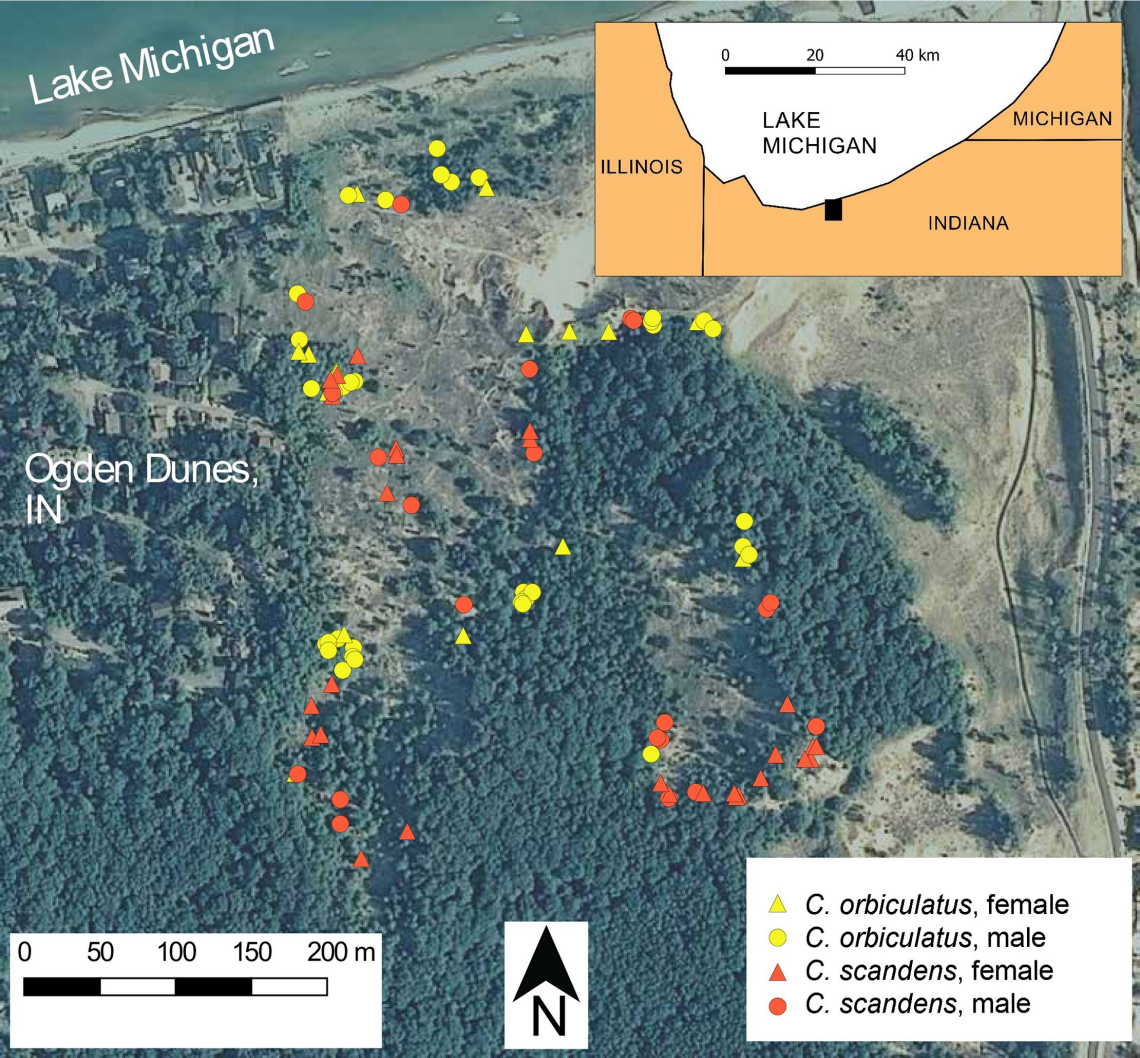
Spatial distribution of focal *Celastrus* individuals at the Portage Lakefront study site, Indiana Dunes National Park, Indiana, USA. Aerial imagery was accessed through the U.S. Geological Survey’s National Map Viewer, and collected by the National Agriculture Imagery Program (U.S. Department of Agriculture) in the year of the study, 2008.

We hypothesized that reproductive interference of *C*. *scandens* occurs in the presence of *C*. *orbiculatus*. We collected data at the Indiana Dunes National Park (Indiana, USA) at a site where *C*. *scandens* and *C*. *orbiculatus* occur in similar abundances, which provided an opportunity to test this hypothesis (Fig 1). Because both species are dioecious, we could specifically assess the relationship between male floral production and reproductive interference. We used experimental hand-crosses to compare the responses of *C*. *scandens* and *C*. *orbiculatus* to heterospecific pollination. For open-pollinated plants in the field, we evaluated the relationship between the availability of conspecific or heterospecific pollen and rates of fertilization and hybridization. Hybrids were identified using previously published genetic markers. Our study was designed to test our hypothesis of reproductive interference between *C*. *orbiculatus* and *C*. *scandens*, understand the causes of asymmetrical pollen flow, evaluate the threat that heterospecific pollination poses to *C*. *scandens*, and advise conservation strategies for an increasingly rare native plant.

## Materials and methods

### Study species

*Celastrus scandens* L. (Celastraceae), commonly known as American bittersweet or American staff vine, is the only member of the genus native to North America [32]. It is a liana (woody vine) usually found in open habitats. Its range extends from southern Quebec to South Dakota, south to western Texas through Georgia. The native range of *C*. *orbiculatus* Thunb., commonly known as oriental or Asiatic bittersweet, is in Korea, Japan, and China [32] where it is found in thickets and lowland slopes. In North America, it can also thrive in shaded habitats that would likely exclude *C*. *scandens* [33]. The North American range of *C*. *orbiculatus* overlaps with much of the range of *C*. *scandens* east of the Mississippi River, with fewer reports west of the Mississippi River. Both species are usually dioecious, although rare individuals and populations displaying monoecy or bisexual flowers are known. In both species, staminate flowers have five stamens. Pistillate flowers have a single pistil, a superior ovary, and three carpels with two ovules each. A single fruit can have up to six seeds. Insects, particularly native bees, pollinate both species [34], with some wind-pollination observed for *C*. *orbiculatus* [35]. Birds are largely credited as the dispersers of the seeds that sit inside the three bright red fleshy arils surrounded by an orange (*C*. *scandens*) or yellow (*C*. *orbiculatus*) capsule that breaks open with first frost [34].

*Celastrus orbiculatus* was introduced as an ornamental vine to the eastern USA in the mid- to late-nineteenth century. The first reports of it escaping into the wild came in the early twentieth century; by the middle of the twentieth century it was recognized as a pest species rapidly spreading in the eastern USA [27, 36]. The species continues to spread, having recently (c. 2010) been observed for the first time in Minnesota. Land managers and foresters consider *C*. *orbiculatus* a troublesome species because it is a strong competitor that crowds out native vegetation [37], negatively affects forestry operation [38], and can alter natural successional trajectories [39]. Notably, *C*. *scandens* has declined in the eastern United States where invasion by *C*. *orbiculatus* is oldest and most extreme [30, 31, 40], although a causal link between the two patterns has not been established. *Celastrus scandens* has been listed as a species of special concern in several states; it may be extirpated in Delaware, while it is listed as a vulnerable species in New York, a species of special concern in Connecticut, a threatened species in Massachusetts, and an endangered species in North Carolina and Rhode Island.

### Study site, sampling, species identification

Experimental crosses and field observations took place at the Portage Lakefront within the Indiana Dunes National Park (near 41.63°N, 87.18°W). The study site is composed of southern Lake Michigan sand dunes and wooded edges, bounded by the town of Ogden Dunes, Indiana, USA to the west and the Port of Indiana to the east (Fig 1). The site was chosen because reproductive individuals of both species are abundant, which is uncommon. We attempted to include all flowering individuals in the observational studies, but some flowering individuals that may have been in nearby residential areas were not included in the study. In total, the study included 39 *C*. *orbiculatus* individuals (22 staminate, 14 pistillate, and 3 putatively monoecious) and 40 *C*. *scandens* individuals (17 staminate, 21 pistillate, and 2 putatively monoecious). This work was permitted by the National Park Service, permit number NH08.5216.

Field identification using reproductive structures [41] was verified by genetic analysis of leaf tissue. Genomic DNA was extracted from 20–25 mg of ground leaf material using the DNeasy Plant Mini Kit (Qiagen) and each individual was scored at five microsatellite loci that are able to distinguish the two species and their hybrids [29]. Microsatellite loci, PCR conditions, and genotyping methods are described in Zaya et al. [29]. Genetic identities were tested using two Bayesian clustering approaches, STRUCTURE version 2.3 and NewHybrids version 1.1 beta [42, 43] using options previously used to identify *C*. *scandens* X *C*. *orbiculatus* hybrids [29]. We used the posterior probability of an individual genotype belonging to a single genetic cluster, *q*, to categorize hybrid seedlings. Seedlings with *q* > 0.85 were categorized as the result of conspecific pollination, while those with *q* < 0.65 were categorized as hybrids. No seedlings had *q* values between these cutoffs. Spatial coordinates were measured with GPS for each individual included in the study. The coordinates were used to calculate the distance to each staminate plant for every pistillate individual.

### Hand pollinations

Experimental hand cross-pollinations were carried out in 2006 to test differences between the species in accepting heterospecific pollen, a possible mechanism behind asymmetric hybridization. Inflorescences on each of 10 pistillate plants, five pistillate plants of each species, were bagged with bridal mesh while still in bud, to exclude pollinators. For each pistillate plant, 25 flowers were hand-pollinated with freshly dehisced anthers from five staminate plants of the other species (five flowers were devoted to each staminate plant). In total, 250 flowers were hand-pollinated. The flowers were observed approximately 10 days later for signs of fertilization, in the form of an ovary that had swelled and not senesced. We compared the rate of successful heterospecific fertilization in each species.

### Floral production, phenology and open pollination patterns

We recorded the total number of open staminate or pistillate flowers for each individual on 15 dates, between 20 May and 12 June 2008. All open flowers on an individual were counted and recorded when possible. Some individuals were too large or climbed too high to count all flowers, in which case a proportional sampling technique was used. For plants covering a large area, we estimated the number of flowers from approximately three square meters, and extrapolated to the ground area covered by the plants as estimated from on-the-ground measurements.

We collected data on fertilization, fruit set, and hybridization rates from individually marked flowers. On each date that plants were observed, we marked up to 10 newly opened flowers on each pistillate plant with colored string tied to the pedicel. Flowers were chosen haphazardly, as attempts to randomize flower selection were prohibitively slow. We did make an effort to select flowers from different places on the pistillate plant, covering its full width and height. We checked each flower for evidence of fertilization 9 to 17 days after it was marked and recorded fertilization success (persistent and swollen ovary) and followed the development of the same flowers through fruit maturation. The flowers on the cyme of *C*. *orbiculatus* are all of equal rank, while the inflorescence for *C*. *scandens* is a panicle with a hierarchical structure. The position on the inflorescence was recorded for each marked *C*. *scandens* flower in order to investigate potential differences in maternal investment and fertilization rate [see 44]. On 5 October and 11 October 2008, we recorded whether the marked flowers had successfully developed into mature fruits and collected those fruits. Resulting seeds (three seeds per ovary) were prepared for germination according to the method described in Young and Young [45] and sowed in the greenhouse at the University of Illinois at Chicago after 90 days of cold stratification. Greenhouse lighting relied on natural sunlight with an approximately 12-hour photoperiod. Greenhouse temperature ranged from approximately 15°C to 25°C. In total, 104 seedlings resulted from the original 716 marked flowers (43 seedlings from 317 *C*. *scandens* flowers, and 61 seedlings from 399 *C*. *orbiculatus* flowers). We genetically tested the seedlings to determine the species identity of the pollen donor using the nuclear microsatellite DNA markers and statistical methods described briefly above and detailed in Zaya et al. [29].

For both fertilization rate and fruit set, we investigated the relationship with several predictors. Analyses were conducted separately for each species. We were most interested in how fertilization and fruit set were related to the availability of conspecific and heterospecific pollen, but we also tested for a relationship with total floral production of the maternal plant and, in *C*. *scandens*, inflorescence position. The sample size of seedlings was too small to test for correlations between hybridization rate and multiple predictors, but the difference in hybridization rate between species was statistically compared. Details of the data analyses are described below.

Fruits collected during the fertilization study were supplemented by additional fruit collections from *C*. *scandens*, with the goal of increasing sample size and statistical power. Emphasis was placed on fruits from the native species because a) no wild hybrids found had a *C*. *orbiculatus* maternal lineage [29], and b) there is more urgency in understanding hybridization in *C*. *scandens*, as it is species of concern in much of its native range. Approximately 250 fruits were collected from 18 *C*. *scandens* pistillate plants. We extracted seeds from these fruits, applied cold stratification, and conducted genetic analyses using leaf tissue from the resulting seedlings [29]. We tested the relationship between hybridization rate and predictors relating to the availability of heterospecific and conspecific pollen (detailed below).

### Data analysis

To test the hypotheses that fertilization rate, fruit set, and hybridization rate differed for open-pollinated *C*. *scandens* and *C*. *orbiculatus*, we used generalized linear models (GLM) with binomial responses. For each test, the explanatory variable was species identity of maternal plant, and the subjects (i.e., units of replication) were the individual maternal plants (fertilization rate and fruit set, n = 40 [23 *C*. *scandens* and 17 *C*. *orbiculatus*]; hybridization rate, n = 19 [nine *C*. *scandens* and 10 *C*. *orbiculatus*]). Monoecious plants were included in these analyses. For analyses of hybridization rates only plants with flowers resulting in at least one seedling were included.

We were interested in the influence of conspecific and heterospecific pollen on the fate of individual flowers (at fertilization and fruit set), and how it differed between species. To explore these relationships, we used generalized linear mixed-effects models (GLMM), with maternal identity as a random factor. The two species were analyzed separately. The subjects of the analysis were individual flowers (for *C*. *scandens*, n = 317 in 23 maternal groups; for *C*. *orbiculatus*, n = 399 in 17 maternal groups). The response variable tested was the binary fate of a flower at the fertilization stage (fertilized or unfertilized) or fruit stage (developed or not developed). Analyses of fruit development only included fertilized flowers. Four explanatory variables were tested as fixed effects: total floral production during the reproductive period, weighted conspecific mating potential, weighted heterospecific mating potential, and inflorescence position (in *C*. *scandens*, only). We constructed candidate models with a maximum of one fixed effect because of significant collinearity among potential explanatory variables. The “weighted mating potential” is a measure that considers both the distance to and floral production of available mates. Greater values indicate higher potential for pollen transfer. It is constructed to be inversely related to the distance to a potential mate, and positively associated with floral production of a potential mate. The weighted mating potential was calculated after Wagenius et al. [46], with some modification:

$$P_{i}=\sum_{j=1}^{n}e^{-\gamma d_{ij}}\;\log_{10}\left(F_{j}\right)$$ (1)

We removed an incompatibility term, *c*_*ij*_, and instead excluded other pistillate plants in the calculation (those which would have *c*_*ij*_ = 0). We weighted the mating potential by log_10_ male floral production, *F*_*j*_, for each *j*-th father on the particular day that a flower was marked. Distance from the *i*-th mother (which carried the flower) to each *j*-th father is *d*_*ij*_. We used the same value for γ, the inverse of the mean pollination distance (13.3 m^-1^), as Wagenius et al. [46], who studied an insect-pollinated self-incompatible plant (*Celastrus* spp. are dioecious).

To test the relationship between hybridization rate in *C*. *scandens* and the availability of conspecific and heterospecific pollen, we used a GLM with binomial response. The subjects of the analysis were individual maternal plants from which seeds were collected and germinated (n = 18, with 259 total seedlings). The response variable was the proportion of successfully germinated seedlings that were hybrids. Four fixed effects were tested: distance to the nearest conspecific male (staminate *C*. *scandens*), distance to the nearest heterospecific male (staminate *C*. *orbiculatus*), log_10_ floral production of the nearest co-flowering conspecific male, and log_10_ floral production on the nearest co-flowering heterospecific male. All fixed effects were included in separate models. Additionally, we tested three two-way interactions (conspecific distance X heterospecific distance, conspecific distance X conspecific floral production, heterospecific distance X heterospecific floral production). A staminate plant was considered as “co-flowering” if it had open staminate flowers on the mean flowering date of the pistillate subject.

To test for species differences in the experimental hand crosses, we used a GLM to compare the rate of successful heterospecific pollination. The GLM was constructed so that fertilization success in heterospecific crosses was the binomial response and species identity of maternal plant was the explanatory variable (n = 10 pistillate plants, five of each species).

All statistical tests were completed using R version 3.6.2 [47]. The GLMM tests were implemented using the *lme4* package [48], and in all cases random intercept models were constructed with maternal identity as the random effect. For each test, we used the Akaike Information Criterion corrected for small sample size (AICc) to select the best model from a group of candidate models. Akaike weights were calculated for each model, which can be interpreted as the probability that a given model is the one that best approximates the data assuming one exists in the candidate set. In all cases a null model, with no fixed effects, was included as a candidate model. The coefficient of determination (*R*^*2*^) was estimated for GLM using the *rsq* package [49, 50] and for GLMM using the pseudo-*R*^*2*^ (*R*^*2*^_GLMM_) [51, 52] from the *MuMIn* package [53].

## Results

### Hand pollinations

In the experimental hand pollinations, 51 of the 125 (41% [s.e. = 4.4%]) *C*. *scandens* pistillate flowers receiving heterospecific pollen were fertilized compared to only three (2.4% [s.e. = 1.4%]) of the *C*. *orbiculatus* flowers. Statistical comparison confirmed the difference between species. The model that included pistillate plant species identity greatly outperformed the null model (ΔAICc = 60.4, Akaike weight > 0.9999).

### Floral production, phenology, and open pollination patterns

**Fig 2:**
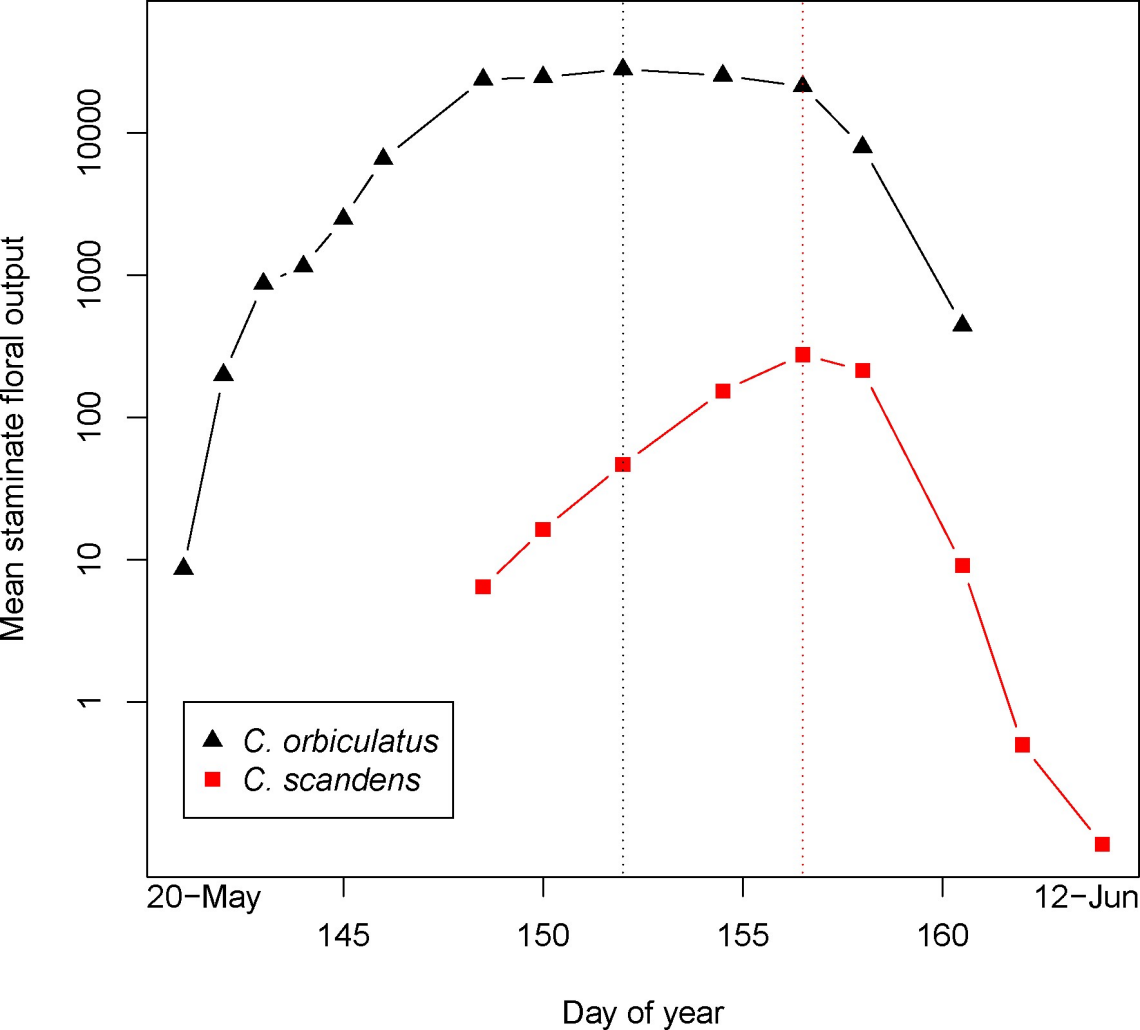
Mean staminate floral production per day. Observations made across 19 staminate individuals in *C*. *scandens* (red squares) and 25 staminate individuals in *C*. *orbiculatus* (black triangles). Vertical dotted lines represent the dates of peak flowering in *C*. *orbiculatus* (31 May, black line) and *C*. *scandens* (5 June, red line).

Observations of floral production show that the invasive *C*. *orbiculatus* has a significant advantage in male and female fecundity compared to the native *C*. *scandens* (Table 1). Pistillate flowering in *C*. *orbiculatus* began on 21 May and ended on 6 June, while in *C*. *scandens* it lasted from 28 May to 12 June. In pistillate flowers, there was a 65-fold difference in output between *C*. *orbiculatus* (mean of 13,100 flowers per plant, s.e. = 4,700) and *C*. *scandens* (202 flowers per plant, s.e. = 50). Staminate flowering in *C*. *orbiculatus* lasted 21 days, from 20 May to 9 June, while in *C*. *scandens* staminate flowering lasted 16 days, from 28 May to 12 June (Fig 2). The peak flowering dates were offset by 5 days—31 May for *C*. *orbiculatus*, 5 June for *C*. *scandens*. Across 15 dates of measurement, we observed 723 flowers per staminate individual in *C*. *scandens* (s.e. = 282) and 142,500 per individual in *C*. *orbiculatus* (s.e. = 43,800), a 197-fold difference. When comparing the respective peak flowering dates, *C*. *orbiculatus* had 277 flowers per staminate individual and *C*. *orbiculatus* had 28,000, more than a 100-fold difference (Fig 2). Even on the peak flowering date for *C*. *scandens* (5 June) there were 77 times more *C*. *orbiculatus* flowers per staminate individual (277 in *C*. *scandens*, 21,200 in *C*. *orbiculatus*). Not surprisingly, these differences in floral production resulted in large differences in conspecific and heterospecific mating potential for each species. For example, conspecific mating potential never exceeded 5.9 for *C*. *scandens* pistillate plants but ranged up to 29.0 for *C*. *orbiculatus* (Fig 2).

**Table 1:** Estimated total annual flower production.

Species	Flower type	N	Flowers per plant (± std. error)
*C*. *scandens*	pistillate	22	202 (± 50)
	staminate	19^1^	733 (± 282)
*C*. *orbiculatus*	pistillate	14^2^	13100 (± 4700)
	staminate	25^3^	142500 (± 43800)

^1^includes one plant that also produced rare pistillate flowers.

^2^includes one plant that also produced staminate flowers.

^3^includes two plants that also produced pistillate flowers.

Three *C*. *orbiculatus* and two *C*. *scandens* individuals were monoecious, with both pistillate and staminate flowers. One type of flower dominated in each individual. In two cases (one of each species) only two pistillate flowers were observed compared to dozens of staminate flowers. In two plants (one of each species) staminate flowers outnumbered pistillate flowers by approximately 3-to-1. In one large *C*. *orbiculatus* plant, there was a 3.5-to-1 ratio in favor of pistillate flowers. Monoecious individuals were included in calculations of explanatory variables (e.g., conspecific mating potential) for other pistillate individuals, but the staminate flowers from the same individual were not considered in such calculations.

**Fig 3:**
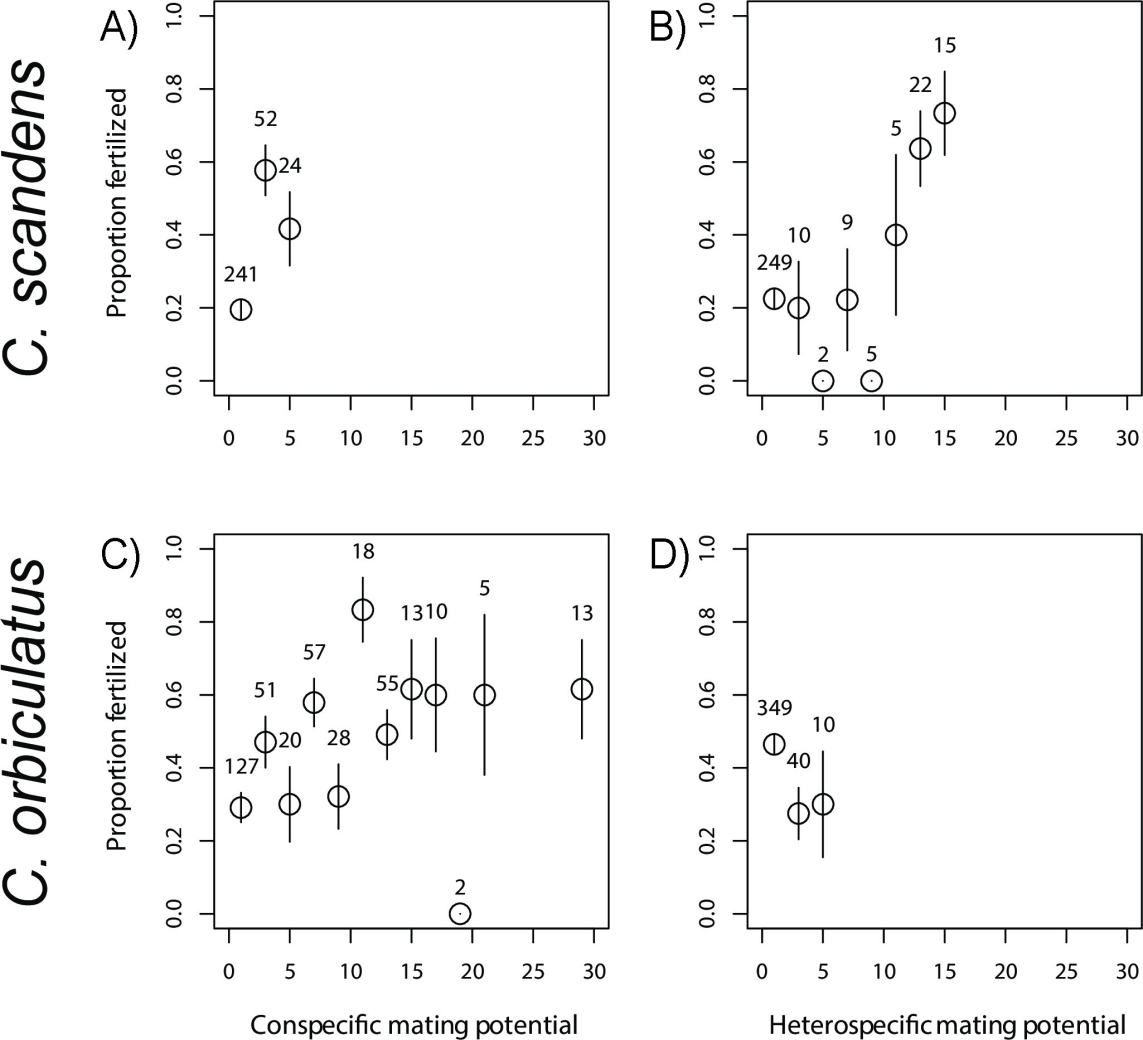
Fertilization rate and conspecific or heterospecific mating potential. Results from 317 individually marked *C*. *scandens* (A, B) and 399 individually marked *C*. *orbiculatus* (C, D) flowers are shown. Numbers above the symbols represent the number of flowers included in each binned category. In *C*. *scandens*, fertilization rate was statistically associated with conspecific mating potential (A; Table 3) and heterospecific mating potential (B; Table 3). In *C*. *orbiculatus*, fertilization rate was only associated with conspecific mating potential (C: Table 4). See Eq 1 for the calculation of mating potentials. Error bars represent the standard error.

Observations on the fate of open-pollinated pistillate flowers are shown in Table 2. Fertilization rate was 27.4% for *C*. *scandens* and 44.1% for *C*. *orbiculatus*. We found statistical support for a difference between species with respect to fertilization rate (GLM that included species as a fixed effect outperformed the null model, ΔAICc = 19.2, Akaike weight > 0.9999, adjusted *R*^*2*^ = 0.130). Conspecific mating potential was associated with increased fertilization in both species (Fig 3) and was the best-supported explanatory variable in both species (Tables 3 and 4). However, for heterospecific mating potential, the patterns differed between the two species. In *C*. *scandens*, heterospecific mating potential was positively correlated with fertilization, a relationship that was strongly supported (ΔAICc = 10.1 when compared to null model, Table 3). In *C*. *orbiculatus*, the relationship was *negative* and weakly supported (ΔAICc = 0.2 when compared to null model, Table 4).

**Table 2:** Fate of open-pollinated pistillate flowers.

	*C*. *scandens*				*C*. *orbiculatus*			
	Plants	N	Observed	Rate (± std. error)	Plants	N	Observed	Rate (± std. error)
Fertilized	23	317	87	0.274 (± 0.026)	17	399	176	0.441 (± 0.025)
Set Fruit^1^	23	81	26	0.321 (± 0.117)	17	176	42	0.239 (± 0.114)
Hybrid Seedlings	9	43	22	0.512 (± 0.167)	10	61	1	0.016 (± 0.040)
Hybrid Seedlings (expanded data)^2^	18	259	101	0.390 (± 0.115)	na	na	na	na

^1^includes only fertilized flowers (two *C*. *scandens* plants with combined 6 marked flowers were removed prior to fruit set).

^2^includes data from additional seedlings germinated from open-pollinated *C*. *scandens* fruits collected at the study site.

**Table 3:** Summary of model selection results for fertilization rate in *C*. *scandens*.

Model	Intercept	Slope	df	*R*^*2*^_GLMM(m)_	*R*^*2*^_GLMM(c)_	AICc	ΔAICc	Akaike weight
conspecific mating potential	-2.04	0.560	3	0.134	0.365	344.1	0	0.622
heterospecific mating potential	-1.53	0.156	3	0.121	0.266	345.1	1.03	0.373
Null	-1.18	N/A	2	0	0.321	355.2	11.08	0.002
total floral output	-1.00	-0.001	3	0.012	0.342	356.6	12.48	0.001
inflorescence position	-1.29	0.029	3	0.001	0.324	356.9	12.80	0.001

Coefficients are at the logit scale. All models are GLMMs that include random intercepts for pistillate plant identity. *R*^*2*^_GLMM(m)_ refers to the marginal pseudo-*R*^*2*^, and *R*^*2*^_GLMM(c)_ refers to the conditional pseudo-*R*^*2*^ [51, 52].

**Table 4:** Summary of model selection results for fertilization rate in *C*. *orbiculatus*.

Model	Intercept	Slope	df	*R*^*2*^_GLMM(m)_	*R*^*2*^_GLMM(c)_	AICc	ΔAICc	Akaike weight
conspecific mating potential	-0.73	0.059	3	0.037	0.200	521.0	0	0.602
total floral output	-0.10	-0.00002	3	0.049	0.289	523.9	2.81	0.148
heterospecific mating potential	-0.23	-0.198	3	0.012	0.222	524.1	3.06	0.130
Null	-0.36	N/A	2	0	0.211	524.3	3.22	0.120

Coefficients are at the logit scale. All models are GLMMs that include random intercepts for pistillate plant identity. *R*^*2*^_GLMM(m)_ refers to the marginal pseudo-*R*^*2*^, and *R*^*2*^_GLMM(c)_ refers to the conditional pseudo-*R*^*2*^ [51, 52].

When following the fate of marked pistillate flowers, we found that 8.4% of flowers in *C*. *scandens* developed into mature fruits (Table 2). In *C*. *orbiculatus*, 10.8% of flowers developed into mature fruits (Table 2). When considering only fertilized flowers, the fruit set rates were 32.1% in *C*. *scandens* and 23.9% in *C*. *orbiculatus*. We did not find statistical support for a difference between species when considering all flowers (GLM with no fixed effect was preferred, ΔAICc = 1.0, Akaike weight = 0.627) or only fertilized flowers (ΔAICc = 0.41, Akaike weight = 0.551).

In *C*. *scandens*, the proportion of fertilized flowers that developed to fruit was not associated with any of the explanatory variables we tested, as the null model outperformed all candidate models with fixed effects (Table 5). In *C*. *orbiculatus*, fruit development was negatively associated with total floral production; fewer fertilized flowers successfully developed on pistillate plants with more flowers (Table 6). The model with heterospecific mating potential had statistical support similar to the null, and the model with conspecific mating potential had substantially less statistical support (Table 6).

**Table 5:** Summary of model selection results for fruit set in *C*. *scandens*.

Model	Intercept	Slope	df	*R*^*2*^_GLMM(m)_	*R*^*2*^_GLMM(c)_	AICc	ΔAICc	Akaike weight
Null	-0.951	N/A	2	0	0.432	98.7	0	0.389
conspecific mating potential	-1.384	0.2106	3	0.020	0.436	100.3	1.63	0.172
inflorescence position	-0.716	-0.06454	3	0.006	0.422	100.5	1.83	0.156
heterospecific mating potential	-0.778	-0.04594	3	0.015	0.424	100.6	1.89	0.151
floral output	-0.960	0.00004	3	0.000	0.431	100.8	2.16	0.132

Coefficients are at the logit scale. All models are GLMMs that include random intercepts for pistillate plant identity. *R*^*2*^_GLMM(m)_ refers to the marginal pseudo-*R*^*2*^, and *R*^*2*^_GLMM(c)_ refers to the conditional pseudo-*R*^*2*^ [51, 52].

**Table 6:** Summary of model selection results for fruit set in *C*. *orbiculatus*.

Model	Intercept	Slope	df	*R*^*2*^_GLMM(m)_	*R*^*2*^_GLMM(c)_	AICc	ΔAICc	Akaike weight
floral output	-0.834	-0.00003	3	0.115	0.178	191.1	0	0.557
heterospecific mating potential	-1.380	0.307	3	0.024	0.150	193.2	2.13	0.192
Null	-1.190	N/A	2	0	0.128	193.5	2.37	0.170
conspecific mating potential	-1.397	0.025	3	0.008	0.135	195.0	3.88	0.080

Coefficients are at the logit scale. All models are GLMMs that include random intercepts for pistillate plant identity. *R*^*2*^_GLMM(m)_ refers to the marginal pseudo-*R*^*2*^, and *R*^*2*^_GLMM(c)_ refers to the conditional pseudo-*R*^*2*^ [51, 52].

Genetic analyses of germinated seedlings that arose from marked flowers showed a large difference in the number of hybrid seedlings between the two species (Table 2). In *C*. *scandens*, 22 of 43 (51.2%) seedlings that germinated were hybrids, while only one of the 61 (1.6%) seedlings from *C*. *orbiculatus* were hybrids. We found strong statistical support for a difference between species with regard to hybridization rate. The GLM that included species as a predictor of hybridization rate performed much better than the null model (ΔAICc = 37.6, Akaike weight > 0.9999, adjusted *R*^*2*^ = 0.419).

**Fig 4:**
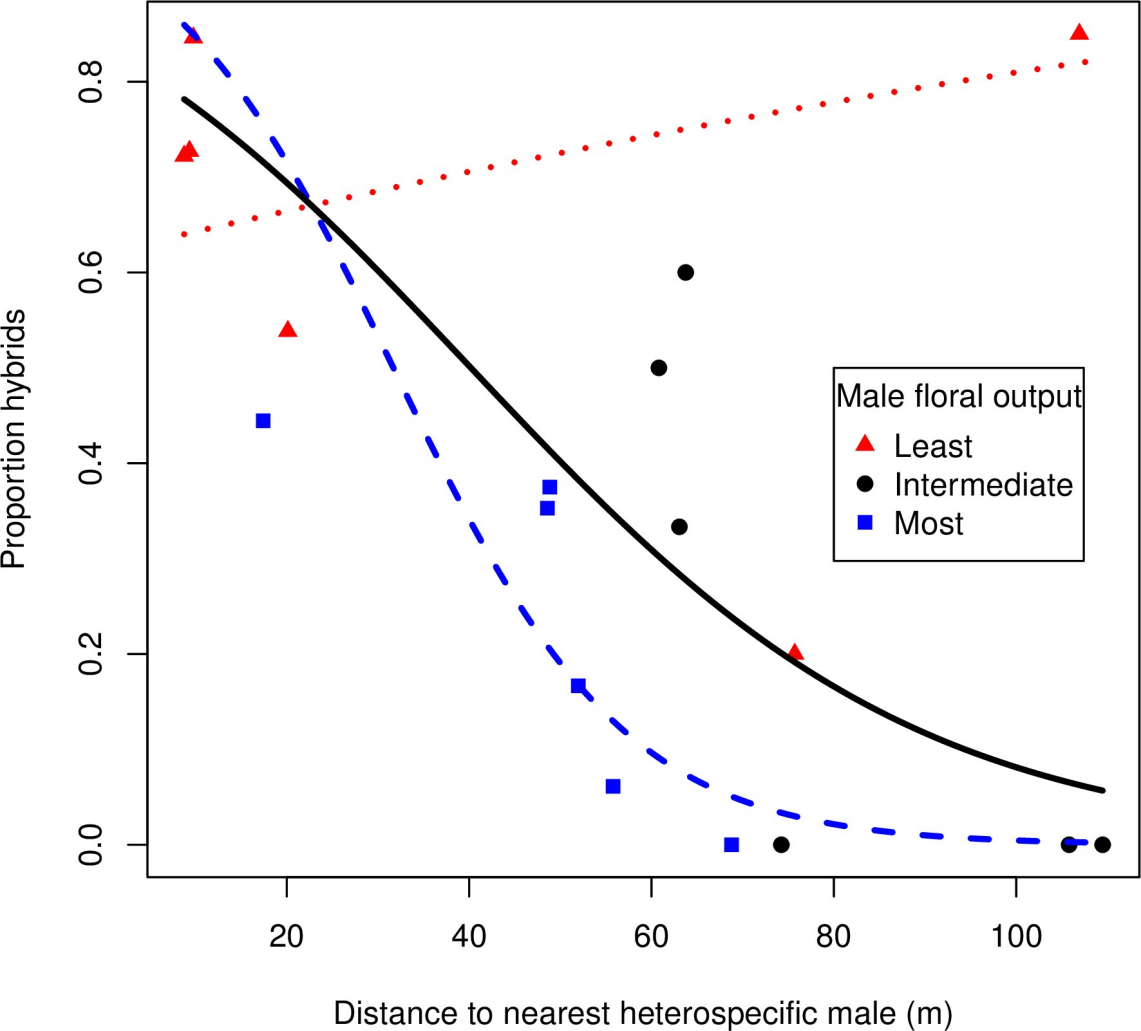
Hybridization rate and distance to the nearest heterospecific male in *C*. *scandens*. Data from open-pollinated pistillate plants. Each point represents the proportion hybrids from a single pistillate *C*. *scandens* (n = 18 pistillate plants, with 259 total seedlings). Red triangles represent the six pistillate individuals for which the nearest *C*. *orbiculatus* had the fewest flowers, blue squares represent the six pistillate individuals for which the nearest *C*. *orbiculatus* had the most flowers. Black circles are pistillate plants for which the nearest *C*. *orbiculatus* had an intermediate number of flowers. Curves represent the predicted values from the best performing GLM, and colors correspond to the points.

When an additional 216 *C*. *scandens* seedlings were included, from 18 pistillate plants, supplementing the data from the individually marked flowers, we found an overall hybridization rate of 0.39 (Table 2). For this expanded data set, the model for best predicting hybridization rate included the distance to the nearest staminate *C*. *orbiculatus*, log floral production of the nearest co-flowering staminate *C*. *orbiculatus*, and the interaction of these two predictors (Akaike weight > 0.9999; Table 7). This model vastly outperformed the next best model, which included only the log floral production of the nearest heterospecific male (ΔAICc = 34.4, Akaike weight < 0.0001). Hybridization rate increased as the distance to the nearest *C*. *orbiculatus* staminate plant decreased (Fig 4). This relationship was more pronounced when the nearest *C*. *orbiculatus* male had greater floral production, as demonstrated by the statistical interaction (Fig 4; Table 7). Models including fixed effects associated with the nearest conspecific male were not well-supported (ΔAICc > 70 compared to top model, Akaike weight < 0.0001), though they performed better than the null model (Table 7).

**Table 7:** Summary of model selection results for hybridization rate in *C*. *scandens*.

Model	Intercept	Slope1	Slope2	Interaction Term	df	*R*^*2*^	AICc	ΔAICc	Akaike weight
1: Dist.Orb	-3.48	0.184	1.387	-0.0611	4	0.844	71.0	0.00	> 0.9999
X									
2: Output.Orb									
1: Output.Orb	3.56	-1.126	N/A	N/A	2	0.484	105.4	34.43	< 0.0001
1: Dist.Orb	0.27	-0.014	N/A	N/A	2	0.128	141.3	70.25	< 0.0001
1: Dist.Scan	0.33	-0.044	N/A	N/A	2	0.100	141.5	70.43	< 0.0001
1: Dist.Scan	0.68	-0.036	-0.012	0.0001	4	0.159	143.1	72.10	< 0.0001
X									
2: Dist.Orb									
1: Dist.Scan	-1.09	0.004	0.632	-0.0208	4	0.114	146.6	75.56	< 0.0001
X									
2: Output.Scan									
1: Output.Scan	-1.17	0.378	N/A	N/A	2	0.046	147.0	76.03	< 0.0001
Null	-0.45	N/A	N/A	N/A	1	0	149.1	78.06	< 0.0001

All models are GLMs with binomial response. Coefficients are at the logit scale. When testing a model with an interaction, both main effects are also included in the model. For models with multiple fixed effects, the order of the slopes corresponds to the order given in the ‘Model’ column. *R*^*2*^ is calculated after [50]. ‘Dist.Orb’ = distance to the nearest staminate *C*. *orbiculatus*, in meters. ‘Output.Orb’ = log_10_ floral output of the nearest staminate *C*. *orbiculatus*. ‘Dist.Scan’ = distance to the nearest staminate *C*. *scandens*, in meters. ‘Output.Scan’ = log_10_ floral output of the nearest staminate *C*. *scandens*.

## Discussion

Our study was designed to investigate the reproductive interactions between a declining native plant, *C*. *scandens*, and an aggressive invasive congener, *C*. *orbiculatus*. *Celastrus orbiculatus* thrives across the range of conditions where *C*. *scandens* occurs [37], creating the potential for interactions with the invasive vine in most places where *C*. *scandens* is found. We chose a study site where both species occur in roughly equal abundances, and we investigated fertilization and hybridization patterns in both species. Our findings confirmed our hypothesis that reproductive interference of *C*. *scandens* occurs in the presence of *C*. *orbiculatus*.

Despite similar abundances of individuals carrying staminate flowers (n = 19 for *C*. *scandens* and n = 25 for *C*. *orbiculatus*), the invasive vine had an extreme advantage in male floral production, producing hundreds more flowers per staminate plant than the native. In *C*. *orbiculatus*, the axillary positioning of inflorescences allows flowers to form along an entire stem*—*as opposed to the terminal inflorescences in *C*. *scandens* that are limited to the end of a stem [30]. An extreme advantage in flower production by invasive species compared to their native relatives seems to be a common trait of invasive plants [18]. The 200-fold advantage for *C*. *orbiculatus* we found in terms of male floral production (Fig 2) is similar to the average reported in a recent meta-analysis [18]. These authors suggest that high floral production of invasive plants allow them to avoid pollen limitation by attracting more pollinators than native species. The invasive species also had an advantage in female floral production, with 65 times more flowers per pistillate plant. Both species had similar rates of fertilization and fruit development in this study, and *C*. *orbiculatus* was found to have greater seed germination rates in other studies [31, 40]. Thus, we also have evidence of a large advantage in female fecundity for the invasive vine. For *C*. *orbiculatus*, fruit set decreased in pistillate plants with greater total output, where the strongest predictor of fruit development was a negative association with total floral production (Table 4). This may indicate that *C*. *orbiculatus* experiences resource limitation at our study site.

The high male fecundity of *C*. *orbiculatus* appears to explain the increased fertilization rate in nearby pistillate plants of both species (Fig 2). The best predictors of fertilization rate differed between the two species. For the native species, both increased conspecific and heterospecific mating potential were correlated with higher fertilization rates. For the invasive species, only conspecific mating potential was significantly correlated with fertilization. Thus, proximity to flowering staminate *C*. *orbiculatus* increases fertilization of both the native and invasive species. These results support an emerging pattern that underscores the threat that heterospecific pollen from invasive plants may pose to native plant reproductive success [12, 17]. In this case, it is likely that high rates of heterospecific pollination lead to usurpation of *C*. *scandens* ovules through production of hybrids. Our previous study found that hybrids show reduced seed set and small, likely inviable pollen [29], so the reproductive effort of *C*. *scandens* is probably wasted on production of hybrid offspring. Early-flowering *C*. *scandens* may be more susceptible to hybridization because of greater exposure to heterospecific pollen as peak flowering in *C*. *orbiculatus* occurs at about the same time as the start of flowering in *C*. *scandens*.

Once a flower is fertilized, none of our potential explanatory variables predicted development to fruit in *C*. *scandens* (Table 5). With regards to the variables we measured, all fertilized flowers have the same likelihood of developing into fruit. Most importantly, we did not find statistical evidence that flowers exposed to large amounts of heterospecific pollen were less likely to develop into fruit (i.e., were more likely to be aborted). This pattern is consistent with the high rates of hybridization we measured in *C*. *scandens* seedlings. Genetic tests of seedlings showed that in *C*. *scandens*, 39% of seedlings tested were hybrids, while hybrids comprised only 1.6% of seedlings from *C*. *orbiculatus*. These values are notable because of the extreme asymmetry (nearly unidirectional) and because such a high proportion of *C*. *scandens* seedlings were hybrids. The proportion of hybrid seedlings in *C*. *scandens* is large compared to some other studies that measured hybridization rates between native and introduced species, such as the 16% and less reported for *Eucalyptus nitens* and *E*. *ovata* in Tasmania [54] or the 30% and less for *Eucalyptus benthamii* and *E*. *viminalis* in New South Wales [55], but it is not unprecedented. In unmanipulated plots, Burgess et al. [56] found 77% of seedlings from *Morus rubra* were hybrids, and Zalapa et al. [57] reported that 55% of elm trees sampled in Wisconsin were hybrids between native *Ulmus rubra* and the invasive *U*. *pumila*.

Interestingly, neither the proximity nor floral production of conspecific males were associated with hybridization rate in *C*. *scandens*. On the other hand, hybridization in *C*. *scandens* was higher when staminate *C*. *orbiculatus* were nearby (Fig 4), and the spatial scale for the impact of heterospecific pollination was relatively large. The best model predicts a hybridization rate of 43% (s.e. = 4.0%) for *C*. *scandens* that were 50 meters from the nearest *C*. *orbiculatus* staminate plant of mean size, and 11% (s.e. = 4.0%) for individuals that were 100 meters away (Fig 4). Native bees thought to be the primary pollinators for *Celastrus* can have flight distances much greater than 100 meters [58]. Our results are comparable to previous work on *Morus* [22, 56]. After removing all introduced individuals and hybrids within 50 meters of the native individuals, Burgess et al. [22] found 63% of seedlings collected from *M*. *rubra* were fertilized by *M*. *alba* or hybrids. We previously reported only 20 hybrids in a widespread sample of 239 (8.4%) non-native *Celastrus* [29]. In our system, the threat posed by heterospecific pollination is not replacement by hybrids or introgression, but rather asymmetric reproductive interference.

Results from the experimental hand crosses showed that *C*. *scandens* readily accepts *C*. *orbiculatus* pollen, but *C*. *orbiculatus* largely rejects pollen from *C*. *scandens*, providing direct evidence that the two species react differently when heterospecific pollen arrives to a stigma. Such differences in prezygotic barriers can be responsible for the creation of asymmetric hybridization [19], and indeed the few *Celastrus* hybrids identified in the field all had a *C*. *scandens* seed parent [29]. The difference in the ability to recognize and reject heterospecific pollen might be expected in this system; the native range of *C*. *orbiculatus* overlaps with several congeners, while *C*. *scandens* is not naturally sympatric with any closely related species [32]. Heterospecific tolerance or avoidance is likely to evolve in regions with higher floral diversification [59] and the degree of tolerance may be related to the recipient’s history of exposure to heterospecific pollen [60]. Pollen germination and pollen tube growth may be difficult to prevent between closely related species because they share similar stigma morphology and pollen-stigma recognition systems [12, 19, 61]. However, invasive species may be especially tolerant of heterospecific pollen. In a coastal dune system investigated by Suárez‐Mariño [17], the invasive species (*Bidens pilosa*) received more heterospecific pollen than the natives (*Cakile edentula* and *Scaevola plumieri*), but heterospecific pollen receipt only had negative effects on the native species. In our system, it is unlikely that *C*. *orbiculatus* received more heterospecific pollen than *C*. *scandens*, but the hand pollination results indicate that the invasive species would be able to reject almost all it received. Thus, differences in male fecundity as well as different responses to heterospecific pollen both contribute to asymmetric hybridization in this system.

Overall, the results of our study strongly suggest that the decline of *C*. *scandens* in its historical range is due at least in part to reproductive interference by *C*. *orbiculatus*. Although *C*. *orbiculatus* has been shown to be a superior competitor in several respects and can escape the negative effects of high density [33, 40, 62], the targeted nature of reproductive interference and the spatial scale of its action make it a more likely explanation than resource competition alone. *Celastrus orbiculatus* is a recent invader of North America that continues to spread and proliferate. The density-dependence associated with reproductive interference through heterospecific pollination suggests that the decline of *C*. *scandens* will accelerate and spread over a larger area, unless large and broad efforts to reverse the spread of *C*. *orbiculatus* are undertaken. A feedback between decreased abundance and increased heterospecific pollination in *C*. *scandens* may occur [63], potentially leading to extirpation from much of its native range. Efforts to conserve *C*. *scandens* can derive guidance from our results, such as reducing exposure to *C*. *orbiculatus* pollen and providing substantial isolation from *C*. *orbiculatus*, as reproductive interference may span tens to hundreds of meters. Previous work showed that hybrids have reduced fecundity [29], so introgression does not threaten the genetic identity of *C*. *scandens* at this time. Thus, *C*. *scandens* populations that were previously exposed to heterospecific pollen may still represent pure lines that are suitable for conservation or re-establishment.
